# Effects of Chronic Alcohol Use Disorder on the Visual Tilt Illusion

**DOI:** 10.3389/fpsyt.2021.647615

**Published:** 2021-07-23

**Authors:** Guoqing Gao, Liangshuang Yin, Jun Cheng, Rui Tao, Yu Liu, Liangjun Pang, Zhengchun Wang

**Affiliations:** ^1^Department of Psychiatry, Affiliated Psychological Hospital of Anhui Medical University, Hefei, China; ^2^Department of Psychiatry, Heifei Fourth People's Hospital, Hefei, China; ^3^Department of Psychiatry, Anhui Mental Health Center, Hefei, China; ^4^Department of Psychiatry, General Hospital for Prison in Anhui Province, Hefei, China; ^5^The Affiliated People's Hospital of Ningbo University, Ningbo, China

**Keywords:** alcohol use disorder, tilt illusion, visual perception, inhibition, primary visual cortex

## Abstract

**Rationale:** Among the serious consequences of alcohol use disorder (AUD) is the reduced ability to process visual information. It is also generally agreed that AUD tends to occur with disturbed excitation–inhibition (EI) balance in the central nervous system. Thus, a specific visual behavioral probe could directly qualify the EI dysfunction in patients with AUD. The tilt illusion (TI) is a paradigmatic example of contextual influences on perception of central target. The phenomenon shows a characteristic dependence on the angle between the inducing surround stimulus and the central target test. For small angles, there is a repulsion effect; for larger angles, there is a smaller attraction effect. The center-surround inhibition in tilt repulsion is considered to come from spatial orientational interactions between orientation-tuned neurons in the primary visual cortex (V1), and tilt attraction is from higher-level effects of orientation processing in the visual information processing.

**Objectives:** The present study focuses on visual spatial information processing and explores whether chronic AUD patients in abstinence period exhibited abnormal TI compared with healthy controls.

**Methods:** The participants are 30 male volunteers (20–46 years old) divided into two groups: the study group consists of 15 clinically diagnosed AUD patients undergoing abstinence from alcohol, and the control group consists of 15 healthy volunteers. The TI consists of a center target surround with an annulus (both target and annulus are sinusoidal grating with spatial frequency = 2 cycles per degree). The visual angle between center and surround is a variable restricted to 0°, ±15°, ±30°, or ±75°. For measuring the TI, participants have to report whether the center target grating orientation tilted clockwise or counterclockwise from the internal vertical orientation by pressing corresponding keys on the computer keyboard. No feedback is provided regarding response correctness.

**Results:** The results reveal significantly weaker tilt repulsion effect under surround orientation ±15° (*p* < 0.05) and higher lapse rate (attention limitation index) under all tested surround orientations (all ps < 0.05) in patients with chronic AUD compared with health controls.

**Conclusions:** These results provide psychophysical evidence that visual perception of center-contextual stimuli is different between AUD and healthy control groups.

## Introduction

Chronic extensive alcohol consumption affects basically all organs, including most brain areas (1–4). Particularly, the primary visual cortex (V1) is vulnerable to any noxious input, such as bisphenol A (5), methanol (6), or organic solvents (7). Alcohol consumption, sporadically or chronically, impairs visual function, as documented in animal research (8–11), human imaging studies (12), and psychophysical measurements (13–15).

Alcohol use disorder (AUD) is characterized by a chronic disorder of alcohol dependence (16). AUD patients who have been craving alcoholic beverage, developing tolerance to the intoxicating effects, and developing neurologic signs of withdrawal when they stop drinking (17–19). The neurotoxic effects of chronic alcohol ingestion on the central nervous system include structural, cognitive, and behavioral dysfunctions (20–23). Moreover, an abnormal excitation/inhibition ratio is associated with ethanol-related cortical deficits in AUD patients (24). It is demonstrated that chronic alcohol consumption increases the number of glutamate receptors and reduces the number of GABA receptors (24). Particularly, the impaired visual processing abilities induced by chronic extensive alcohol consumption are explained by altered metabolism in the primary visual cortex (12), impaired brain electrical activities (25), and reduced activation of occipital areas (26).

**Figure 1:**
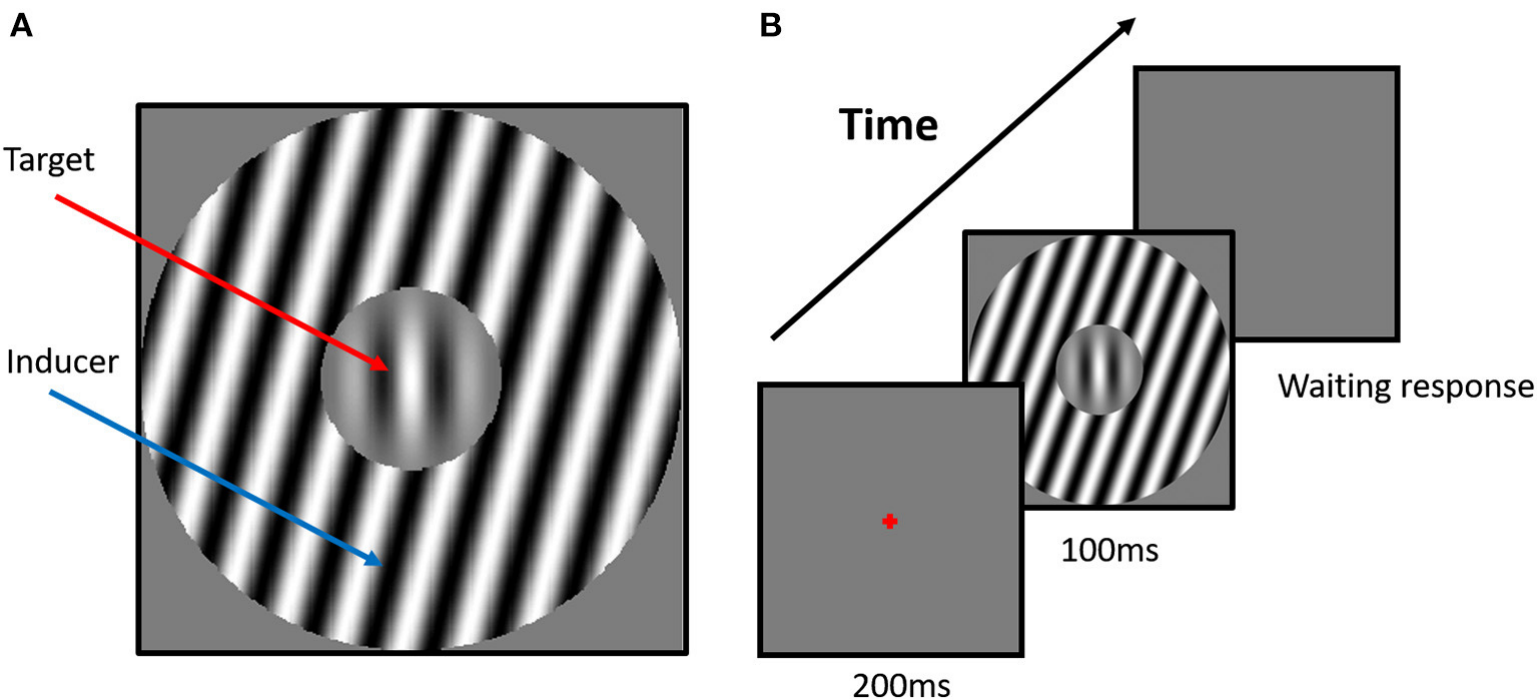
Example stimuli and experiment scenario. **(A)** The stimulus configuration for the tilt repulsion condition. In this case, the orientation of the surround inducer is +15°, and the central target is oriented vertically (0°), which induces a repulsion in the perceived orientation of the center grating, now appearing to be tilted left of vertical. **(B)** The scenario of the tilt illusion (TI) experiment. The classical one-interval task was used in the current experiments. The surround orientation could have one of seven values (−75°, −30°, −15°, 0°, +15°, +30°, +75°) relative to the orientation of the center grating, and we manipulated the center orientation in each trial with respect to vertical according to the participant's responses to measure each subject's perceived vertical.

**Figure 2:**
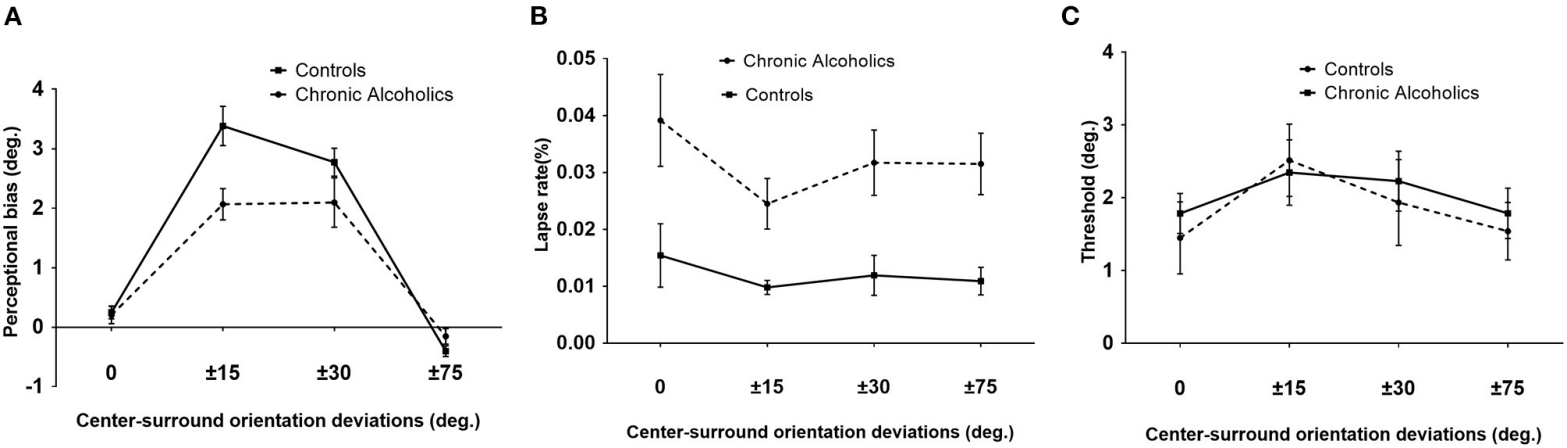
Tilt illusion (TI) results. **(A)** TI effects, indicated by perceptual orientation bias necessary to perceive the center as vertical, as a function of various angles between surround inducer and center target for patients with chronic alcoholism (broken line) and healthy controls. Perceptual biases of ±15° and ±30° are tilt repulsion effects, while those under ±75° were attraction effects. **(B)** Lapse rate values for chronic alcoholics (broken line) and controls. **(C)** Orientation thresholds around perceived verticality for chronic alcoholics (broken line) and controls (blue). Error bars are SEM.

The perceived orientation of center target was biased by the simultaneously presented surround stimulus (Figure 1A), a phenomenon known as tilt illusion (TI) (27). Particularly, the physical orientation of center target was strongly misperceived by participants when the surround orientation had an angular difference between 0° and 50° (repulsion effect), while a systematic weaker effect was observed when angular difference was around 75° (attraction effect) (example in Figure 2A) (28, 29). Lateral inhibition between neurons tuned to different orientations at the same location, as well as between those tuned to the same orientation at different locations, is proposed as the neural mechanism of repulsive and attractive TI (28, 30, 31). The TI has been proven a valuable tool in establishing the extent of unconscious processing in human visual cortex and in investigating the degree of cue invariance with which orientation is processed (27).

Given that AUD patients suffer from disturbed excitation/inhibition balance (24), for example, increased glutamate receptors and reduced GABA receptors (24), we expect that AUD patients should possess decreased overall inhibition. We investigate this hypothesis by using the center-surround TI scenario as a probe of inhibition changes, such that the weakened inhibitory system should decrease the magnitude of TI.

## Materials and Methods

### Subjects

The study includes 15 AUD subjects and 15 healthy individuals. All study participants are male between 20 and 46 years of age. The alcohol-dependent subjects are recruited from the Anhui Mental Health Center and meet the criteria for alcohol dependence (Table 1). All subjects are examined by an ophthalmologist before psychophysical testing. No subject has anatomical abnormalities that could be detected by ophthalmological examination. This is particularly important for the chronic AUD subjects because permanent damage to the papillomacular bundle can occur in advanced forms of alcoholism, which can be detected by retinoscopy (32).

**Table 1:** Demographic and alcohol-related data for patients and controls.

	**Alcohol-dependent subjects**	**Healthy subjects**
N	15	15
Age, years	36.53 ± 1.74	33.93 ± 1.52
Education, years	8.80 ± 0.64	14.87 ± 1.03
Left eye (logMAR)^a^	0.09 ± 0.05	0.03 ± 0.03
Right eye (logMAR)	0.04 ± 0.03	0.04 ± 0.03
Alcohol consumption^b^	195.90 ± 21.31	/
Abstinence, days^c^	31.73 ± 2.95	/
Age in years at first drinking	17.93 ± 0.92	/
Age in years at onset of dependence	27.13 ± 1.59	/
Duration of dependence, years	10 ± 1.58	/

a *LogMAR indicates the logarithm of the minimum angle of resolution*.

b *Alcohol consumption was defined as grams of pure alcohol per day preceding detoxification*.

c *The abstinence for these subjects was calculated from the date of admission to the hospital to the date of the study. Data are expressed as mean ± SEM*.

Patients are included in the present study if they fulfilled the following criteria: (1) current diagnosis of AUD as defined in the Diagnostic and Statistical Manual of Mental Disorders, Fifth Edition (DSM-V); (2) normal or corrected to normal visual acuity (20/20 visual acuity) and no history of past or present ocular or neural diseases that could affect visual functions; (3) no current blood alcohol and alcohol misuse maintained until hospitalization (in order to ensure that all patients are tested within their first month of detoxification, most of the subjects are abstinent for more than 20 days before the date of the study). Patients are excluded if they (1) are diagnosed with a disorder in the psychotic spectrum or (2) regularly used other addictive substances (except nicotine).

Control participants are recruited from the local community, who have normal or corrected to normal visual acuity (20/20 visual acuity) and no report of current or past history of alcoholism, neurological and/or psychiatric disease, or medication. None of them presents a personal or family history of chronic alcoholism. Alcohol-related data of patients and controls are shown in Table 1.

### Ethics Statement

This research has been approved by the ethics committee of the Mental Health Center of Anhui Province. All participants are provided with informed consent forms before taking part in the psychophysical assessment, which followed The Code of Ethics of the World Medical Association (Declaration of Helsinki) for experiments involving humans.

### Equipment and Stimulus

The visual stimuli are displayed on a 17-inch CRT monitor (Sony G520, Sony Corporation, Tokyo Japan; 85 Hz, resolution of 1,280 × 960 pixels) and generated by self-programmed Matlab functions (MathWorks Inc.) with PsychToolBox-3 extensions (33). The original 8 bits per pixel luminance range digitization is extended above 10 bits with the contrast box switcher (34), and the monitor is calibrated daily with a custom laboratory automated procedure.

A chair is set at 200 cm from the video screen, with a support for the chin and forehead to control the distance, and the stimuli are viewed binocularly. A black cardboard with a 30-cm-diameter circular window is delimited in front of the monitor to avoid any local cues of the vertical/horizontal position (35). All tests are performed in a dark room with 0.01 cd/m^2^ of background luminance. The stimuli used to measure the TI are defined by center-surround configuration with a central Gabor patch (target) surrounded by an annulus of the sine-wave grating (inducer). The Gabor patch is defined as previous reports (35, 36).

The stimulus in each trial is presented for nine frames (~100 ms) after 17 frames (~200 ms) fixation, and no feedback is provided regarding the response correctness (Figure 1B). The orientation of the inducer is defined with respect to the orientation of the central target and is one of seven predefined values (−75°, −30°, −15°, 0°, +15°, +30°, +75°). Particularly, a 0° inducer indicates that the orientation of the inducer has the same orientation as the central target. Positive and negative values corresponded to clockwise and counterclockwise orientations from 0°, respectively (Figure 1). There are 280 trials (40 trials × 7 surround orientations) in the orientation discrimination task, and all conditions are pseudo-randomly presented to each subject. The target orientation is altered across the trials to estimate each subject's perceived upward orientation of the target under a surround orientation. A weighted up–down adaptive procedure is used for psychometric curve measurement. For each surround orientation, two staircases are assigned with up/down steps of 3/1 and 1/3 in steps of 1°, respectively. Each staircase contains 20 trials, with a starting direction of −21°/+21° positioned at the opposite side of the convergence point, which allows for rapid measurement within the transition region of the psychometric function. All stimuli are achromatic and are presented in real time at the center of the screen.

### Psychophysical Procedures

Before the examination, the trial procedures and aims are clearly and carefully explained to each of the subjects. Each participant undergoes an ophthalmological visual examination and answers questionnaires with demographic questions (information about age, gender, education, duration of abstinence and dependence, alcohol consumption, age at first drinking, and onset of dependence).

Experiments are initiated by subjects with a predefined keyboard press. A small red dot in the center of the CRT is provided as a fixation point on which observers are to hold their gazes. After the stimulus disappeared from the screen, participants have to report whether the target orientation is tilted clockwise or counterclockwise from his internal vertical upward orientation by pressing corresponding keys (right or left arrows) on the computer keyboard. Each observer has a practice session prior to the collection of actual experimental data. That is, a few easy trials (strong target tilts) are conducted to ensure that each observer could understand and perform the trial accurately. The duration of the visual tilt procedure is about 10 min.

### Data Analysis

The magnitude of the TI at each surround orientation is determined by the angular difference between the perceived and physical orientations. The raw data of each surround orientation and condition is fitted with a logistic function that consisted of the proportion of clockwise responses as *p*_*i*_ = *y*_*i*_/*n*_*i*_, where *n*_*i*_ is the number of occurrences under the current target orientation (*x*), and *y*_*i*_ is the number of clockwise responses. Thus, the psychometric function is:

$$\begin{array}{l} \text{p}\left(x\right)=l+\frac{1-2l}{1+\exp\left(-\frac{log\left(21/4\right)}{\sigma }\left(x-\mu \right)\right)} \end{array}$$

where *l* is the subject's lapse rate, μ and σ are the perceived vertical orientation (also called “bias”) for the given surround orientation and the threshold of the subject for perceiving a deviation from verticality, respectively. The “perceptual bias” corresponds to the perceived vertical reference direction (midpoint) in a given surround condition. The discrimination threshold describes the deviation from the seen reference value for reliably (above p = 0.84) seeing a deviation from the perceived reference. The function is adjusted to the data using Bayesian fitting. Prior parameters are: *l*–beta probability distribution with parameters Beta (1.2, 15); σ—gamma probability distribution with parameters Gamma (2.5, 2.5); and μ has a uniform prior. The perceptual biases of a given block of measures are adjusted to a mean of zero by subtracting the average. The perceptual bias (μ), threshold (σ), and lapse (*l*) are extracted using the above methods for each subject, surround direction, and condition.

### Statistical Analysis

The differences between patients with alcoholism and healthy controls are analyzed using *t*-test for age, education, and visual acuity. For the data of the magnitudes of repulsion, a repeated measures analysis of variance (ANOVA) is calculated using “group” (patients–controls) as the between-subject factor and “surround orientation” (0°, ±15°, ±30°, and ±75°) as the within-subject factor. We also perform Bonferroni *post-hoc* multiple comparisons for the repulsions of each test's orientation. All statistical levels used Geisser–Greenhouse epsilon hat when appropriate. Data are expressed as mean ± SE.

## Results

Basic demographic information such as age, education, and visual acuity (VA) was collected for all participants and alcohol-related data for patients with chronic alcohol misuse (Table 1). All subjects had normal visual acuity or decreased visual acuity that could be corrected to normal using spectacle lenses with appropriate dioptric values, and there was no significant difference [*F*_(3, 56)_ = 0.59, *p* = 0.62] in the VA. No significant difference between the groups was observed in age (*t* = 1.13, df = 28, *p* = 0.27). Higher levels of education (*t* = 5.04, df = 28, *p* < 0.0001) were observed in controls compared with AUD patients. The experiment was conducted 3 weeks later of monitored alcohol abstinence for chronic AUD, and the average duration of abstinence was 31.73 ± 2.95 days. The abstinence for these subjects was calculated from the date of admission to the hospital to the date of the study. Age in years at first drinking and age at onset of alcohol dependence were 17.93 ± 0.92 years and 27.13 ± 1.59 years, respectively. By questionnaire investigation, patients with alcoholism reported a mean daily consumption of 195.90 ± 21.31 g/day alcohol during the last month before admission to the hospital and a mean alcoholism history of 10 ± 1.58 years (Table 1).

### Decreased Tilt Repulsion in Patients With Chronic Alcoholism

A 2 × 4 repeated ANOVA with group (chronic AUD and controls) as the between-subjects factor, surround orientation (0°, ±15°, ±30°, and ±75°) as the within-subject factor, and education as the covariate was conducted. The results revealed that there were significant main effects of surround orientation on TI [*F*_(3, 81)_ = 8.25, *p* < 0.001, $\eta_{p}^{2}$ = 0.23] and a significant interaction between surround orientation and group [*F*_(3, 81)_ = 3.17, *p* = 0.05, $\eta_{p}^{2}$ = 0.11]. This interaction effect was driven by a reduced (*p* = 0.03, $\eta_{p}^{2}$ = 0.16) tilt repulsion in chronic AUD under ±15° surround orientation compared with controls (Figure 2A).

### Increased Lapse Rate in Patients With Chronic Alcoholism

The lapse rate of an observer indicated an overall attentional state the observer paid to the current task, which was suggested as an indicator of plausible attentional limit changes or deficits. There was no statistical difference between various surround orientations [*F*_(3, 81)_ = 0.61, *p* = 0.52, $\eta_{p}^{2}\;=$ 0.02] or interaction effects [*F*_(3, 81)_ = 1.32, *p* = 0.27, $\eta_{p}^{2}$ = 0.05]. However, a significant main effect of group on the lapse rate [*F*_(1, 27)_ = 6.67, *p* = 0.016, $\eta_{p}^{2}$ = 0.2] was observed. Bonferroni posttests revealed a higher lapse rate of patients with alcoholism in 0 (*p* < 0.01), ±30° (*p* < 0.05), and ±75° (*p* < 0.05) conditions compared with healthy controls (Figure 2B).

### Similar Orientation Discrimination Performance in the Two Groups

The discrimination threshold described the deviation of the orientation from the perceived verticality in which the participant reported reliable deviation in 84% of trials. The deviation indicates the difficulty of discriminating two close orientations of the center target. Higher deviation values reflect a worse discrimination ability of the participant. The average thresholds for each group were presented in Figure 2C. There was no distinction in orientation discrimination thresholds among various conditions [*F*_(3, 81)_ = 1.81, *p* = 0.17, $\eta_{p}^{2}$ = 0.06] and between groups [*F*_(1, 27)_ = 0.03, *p* = 0. 86, $\eta_{p}^{2}$ = 0.001]. There was no interaction overall [*F*_(3, 81)_ = 0.22, *p* = 0. 80, $\eta_{p}^{2}$ = 0.008] (Figure 2C).

## Discussion

The present study investigated the changes in inhibitory mediated TI in abstinent individuals with AUD vs. healthy controls using human psychophysiological measures. Results showed an obvious center-surround interaction in both the patient and control groups. Patients with chronic AUD had no significant difference in the tilt attraction compared with healthy controls, and the threshold values between two groups were similar. However, there was a weaker tilt repulsion effect in individuals with AUD compared with healthy controls, which was demonstrated by the decreased magnitude of tilt repulsion in the chronic AUD patients compared with those of matched controls. Additionally, there was a significantly elevated lapse rate (attention limitation index) in patients with chronic alcoholism compared with healthy controls in all conditions. The current findings provided evidence for the detrimental effect of alcohol dependency on the early visual information processing.

One of the most important aspects of our results was that it allowed to rule out explanations of reduced repulsive TI in chronic AUD patients due to higher-level effects of orientation processing. TI patterns were systematically modulated by surround orientations consistently across all conditions, which meant participants, both chronic AUD patients and healthy controls, reliably represented individual perceptual sensitivities under all conditions. The decrease in perceptual bias only occurred at surround orientations of 15°, while the attractive TI effect was unchanged, which meant there was abnormal visual processing in early levels of visual processing and perception, while these deficits such as more global, higher-order orientation processing were not visibly affected in chronic AUD patients compared with healthy controls. Another was that the current findings allowed to discard specific explanations of reduced repulsive TI in chronic AUD patients due to attentional changes targeting the exact conditions where the repulsive TI appears. The lapse rates globally increased across all surround orientations and indicated that subjects had global changes in attention to the task, and these “high cognitive” effects were unrelated to specific surround conditions. In other words, deteriorated cognition, that is, attention, represented generalized effects.

Several population changes of center orientation tuning characteristics could contribute to the observed TI effect: amplitude inhibition, tuning width change, shift of neuronal preferred orientation, etc. (37, 38). Additionally, by comparing human psychophysics and neurophysiology (39), TI effects involved two spatial mechanisms: one narrowly tuned orientation that was spatially restricted and the other broadly tuned that was spatially widespread. We inferred, for the moment, that the reduced repulsive TI effect in AUD patients came from either broader orientation tuned neuron populations or a weaker surround amplitude of inhibition, until further evidence is available. Future work on training AUD animal model to perform TI task while simultaneously recording single-unit activities in V1 would clarify the present position.

Several neurotransmitter systems [e.g., gamma-aminobutyric acid (GABA), glutamate, dopamine, acetylcholine, and serotonin systems] were vulnerable to effects of alcoholism. Disrupted GABA, the major inhibitory neurotransmitter, might contribute to the deficits in the V1 inhibition that we observed. Available evidence suggested that acute alcohol potentiated GABA's effects (i.e., it increases inhibition, and often the brain became mildly sedated). However, prolonged and excessive alcohol ingestion reduced the number of GABA receptors. When the person discontinued drinking, decreased inhibition combined with a deficiency of GABA receptors might contribute to overexcitation throughout the brain, including V1. GABAergic system was a major determinant of the level of activity in V1. In the current study, the participants in the patient group were chronic alcohol-dependent people on withdrawal for about 20 days, which meant that the disturbed balance between the inhibitory and the excitatory still exists even during abstinence for about 3 weeks.

It was reported that GABA enhancers ameliorated ethanol withdrawal reaction, which suggested that the GABAergic system is one of the key targets for alcohol toxicity (40). Additionally, levels of GABA(A)-benzodiazepine receptor were reduced in alcohol dependency in the absence of gray matter atrophy (41). Together with current findings of weaker tilt repulsion in chronic AUD, ethanol and GABA interplay might modulate a visual cortical dysfunction in such subjects (11). The inhibitory function was considered to play an important role in the tilt repulsion; the findings of weaker contextual modulations of orientation in chronic alcoholism might imply an altered inhibitory processing in orientation-sensitive neurons in the primary visual cortex (V1) in alcoholism.

There was a significantly elevated lapse rate (attention limitation index) in patients with chronic alcoholism compared with healthy controls. Using the visual probe task, Sinclair et al. (42) explored attentional biases to alcohol, depression, and anxiety related cues and found the reduced attention to depressive and anxiogenic material in abstinent patients. Previous studies suggested an attentional bias toward alcohol-related stimuli at the cost of other stimuli in problematic drinkers (43–45). Research suggested that these stimuli not only attracted attention but that problematic drinkers also found it difficult to disengage their attention from them. Melaugh McAteer et al. (46) investigated both adolescent and adult social drinkers and found comparable alcohol attention bias between them. Recent studies identified abnormal cortical thickness in the superior frontal gyrus, lateral orbitofrontal cortex, and transverse temporal gyrus in alcohol dependence (47). The current findings that increased lapse rate in patients with chronic alcoholism might suggest abnormal attention function in patients with chronic alcoholism.

The “perceptual bias” corresponded to the perceived vertical reference direction (midpoint) in a given surround condition, while the discrimination threshold described the deviation from the seen reference value for reliably (above *p* = 0.84) seeing a deviation from the perceived reference. The deviation indicated the difficulty of discriminating two close orientations of the center target. Higher deviation values reflected a worse discrimination ability of the participant. No difference in orientation discrimination thresholds between the two groups indicated that the difficulty of discriminating two close orientations of the center target was similar between the two groups. The lapse rates indicated subjects' attention to the task. Our results revealed that the lapse rates of AUD patients globally increased across all surround orientations, which indicated that these “high cognitive” effects were unrelated to specific conditions.

Previous studies pointed to an impairment of visual functions caused by alcohol toxicity. Consistently, our results showed a significant decline in amplitude of the tilt repulsion in patients with chronic alcoholism. The latter seemed to be more likely due to the dramatic effects of chronic alcohol ingestion in inhibitory system.

## Data Availability Statement

The original contributions presented in the study are included in the article/supplementary material, further inquiries can be directed to the corresponding author/s.

## Ethics Statement

The studies involving human participants were reviewed and approved by the ethics committee of the Mental Health Center of Anhui Province, Declaration of Helsinki. The patients/participants provided their written informed consent to participate in this study.

## Author Contributions

The measurements were carried out by ZW and GG. ZW, GG, and LY performed the data analysis. ZW and LP contributed to the supervision of data analysis and to the manuscript writing. Research and study design were carried out by all authors.

## Conflict of Interest

The authors declare that the research was conducted in the absence of any commercial or financial relationships that could be construed as a potential conflict of interest.

## Publisher's Note

All claims expressed in this article are solely those of the authors and do not necessarily represent those of their affiliated organizations, or those of the publisher, the editors and the reviewers. Any product that may be evaluated in this article, or claim that may be made by its manufacturer, is not guaranteed or endorsed by the publisher.
